# A Current Update on the Rule of Alternative and Complementary Medicine in the Treatment of Liver Diseases

**DOI:** 10.1155/2013/321234

**Published:** 2013-09-10

**Authors:** Yong-Song Guan, Qing He

**Affiliations:** ^1^Department of Oncology, West China Hospital of Sichuan University, Chengdu 610041, China; ^2^State Key Laboratory of Biotherapy, West China Medical School, Sichuan University, Chengdu 610041, China

## Abstract

There is a vast body of knowledge which is ever-increasing about the treatment of liver disease with alternative and complementary medicine for which hundreds of thousands of literatures have been documented. Liver disease is a general term. This term covers all the potential problems that cause the liver to fail to perform its specified operations. Liver disease has a variety of presentations and causes a great public health problem worldwide which threatens the wellness of billions of people. Incidences of many types of liver disease are currently rising. Although there is still a debate about the entity of alternative and complementary medicine, it is now widely used and it is improving. And it covers the shortages and compensates for the weaknesses of conventional methods in the treatment of liver diseases. Alternative and complementary medicine for liver diseases provides benefits by regulating immunity, controlling disease progression, improving quality of life, and prolonging survival. This paper reviews the increasing interest and growing research into alternative and complementary medicine for liver diseases, with a look at the rough classification, principle of management, evidence-based applications, and issues for prescription and perspectives.

## 1. Introduction

Liver disease is an expression for any damage that reduces the functioning of the liver. This general term covers all the potential problems that cause the liver to fail to perform its specified operations [1]. Liver diseases cause an enormous global public health problem which threatens the wellness of billions of people [2]. More seriously, incidences of many liver diseases are currently rising [3]. Increased immigration, fast and frequent travel, and globalization in the modern world lead to wide spread of virus hepatitis [4]. Obesity produces steatohepatitis which is evolving into a new and major health problem [5]. Heavy alcohol consumption, which is becoming a tendency in younger people, results in steatosis, alcoholic hepatitis, and cirrhosis [6]. Globally, the number of people chronically infected with hepatitis B virus (HBV) has reached 400 million and up to 2 billion people are with the evidence of exposure. Approximately 3% of the world population is infected by HCV or about 170 million people are HCV-infected [7, 8]. The liver is a very complex organ that is essential for living. It is complicated in both structure and function. The liver functions as a factory for products, a storehouse for supply, and a source of various substances for systemic operation and regulation of other organs and tissues [9]. These factors imply that the treatment of liver diseases is complicated and full of difficulties. Management of liver diseases should not be simplified, and one should concern all the aspects of the diseased parts, with the overall situation of the liver being considered [10, 11]. A comprehensive but pinpoint therapy is ideal. However, there are conflicts between the therapy with comprehensive approach and a pinpoint goal [12]. Alternative and complementary medicine for liver diseases has a very broad field that is different from Western or allopathic medicine [13]. This paper reviews the increasing interest and growing research into alternative and complementary medicine for liver diseases, with a look at the rough classification, principle of management, evidence-based applications, and issues for prescription and perspectives. 

## 2. Overview of Liver Diseases

Appropriate and correct classification of liver diseases is the essence for directional and individualized treatment. However, clinically liver diseases are often insidious or present varied symptoms and signs, and this makes the classification of them become difficult and multiplex [39]. Based either on the evidence of their etiology or on the prominent clinical manifestations, liver diseases can be divided briefly into the following categories including alcoholic, autoimmune, cirrhotic, congenital, infectious, intoxicated, metabolic, and space-occupying liver diseases. Some clinical features overlap among the categories, and a disease entity can shift from one category to another [40]. Sometimes there is no clear-cut outline for a disease to fall into a certain category, and a patient may suffer at the same time several liver diseases in different categories [41]. The most well-known example is the gradual transformation from viral hepatitis to cirrhosis then to liver cancer [42]. 

Alcoholic liver diseases are predominate in Western countries, and they are caused by excessive consumption of alcoholic beverages. Alcohol abuse leads to liver disorders characterized by damage of liver cells and consequent liver steatosis and alcoholic cirrhosis [14]. Poor nutrition is often observed in patients of this category. The patients have malabsorption and maldigestion of nutrients and replace alcohol for normal food. The level of malnutrition is correlated to the severity of liver disease. Enteral nutrition can be taken as complementary and alternative medicine. It combats malnutrition and enhances clinical outcome, thus may be considered as frontline therapy [15].

Autoimmune liver diseases are diseases of unknown causes and are reported to be on the increase all over the world. They are concluded when a series of tests suggest that the body's immune system attacks the liver. This category of diseases includes autoimmune hepatitis, primary biliary cirrhosis, and primary sclerosing cholangitis [16]. When the combination of biochemical, autoimmune, and histological studies implies the category and other categories are excluded, the diagnosis can be established. Standard therapy is the use of prednisone or prednisone with azathioprine. And budesonide may be an additional treatment option for patients without cirrhosis [17]. Curcumin is now used as a supplement in several countries, based on the favorable evidence from more than 65 human clinical trials [18]. Some herbs-induced liver injuries can be autoimmune like, and establishment of causality is necessary [43].

Cirrhosis is a slow process in which healthy liver tissue is replaced gradually with scar tissue. All the inflammatory, alcoholic, and autoimmune liver diseases can end in cirrhosis [44]. The etiology is different and with dynamic change in different areas in the world due to drug use, migration, and lifestyle change. Some cases are idiopathic, for which attention should be paid to a detailed history of drug and food intake to differentiate drug-induced chronic hepatitis, which falls into the category of intoxicated liver diseases [45]. Serious complications occur in patients with severe cirrhosis of the liver, including ascites, esophageal variceal hemorrhage, hepatic encephalopathy, and hepatorenal syndrome. Hemorrhagic ascites seems to be a symbol for poor outcome and remains a challenge to both conventional and alternative medicine [46]. 

Congenital liver diseases are inherited disorders that are present at birth, such as Alagille syndrome, alpha 1-antitrypsin deficiency, galactosemia, hematochromatosis, porphyria, tyrosinemia, type I glycogen storage disease, and Wilson's disease [47, 48]. Some of the ductus malformations are frequently associated with cardiac and other vascular anomalies, and a complex and combined surgery or hybrid operation is needed for treatment [25].

Infectious liver diseases are resulted from various infection, of the liver by different organisms and are more common in the developing countries. These include hepatitis viruses, bacteria, and parasites [28]. Geological difference exists. For example, high rates of hepatitis C virus infection are reported in Egypt while those of hepatitis B virus infection are reported in Asia. Diseases of this category present in the liver such as viral hepatitis, bacterial abscess, tuberculosis, hepatic schistosomiasis, fascioliasis, amebiasis, and echinococcosis. Specific probiotics are shown to have anti-inflammatory and selective antimicrobial effects as supplemental treatment for several acute and chronic infectious processes [30].

Intoxicated liver diseases are caused by toxicity from drug and food intake [32]. Substances toxic to the liver can also be inhaled or absorbed through the skin, such as carbon tetrachloride, formaldehyde, phosphate, and phenylamine [49]. This condition is often encountered in industrial or mining areas. Both acute and chronic intoxication by substances can lead to liver failure [33, 50]. Essential phospholipids from soybean accelerate the improvement or normalization of subjective symptoms in hepatic intoxication [34]. 

Metabolic liver diseases are caused by disorders in metabolism of amino acid, bile acid, carbohydrate, or copper. Nonalcoholic fatty liver disease is the most common liver disease in developed countries, being the hepatic manifestation of the metabolic syndrome [51]. Clinically, laboratory studies demonstrate that either the liver has no structural abnormalities but obvious functional disturbances or the liver cells are injured showing apoptosis, necrosis or cirrhosis [52]. Hepatomegaly can manifest presenting hepatic storage of lipid, glycogen, or other products. Weight reduction is recommended in obese patients with diet as the primary treatment [35].

Space-occupying liver diseases imply massive hepatic lesions that consist of abnormal or nonhepatic contents. These lesions replace the normal tissue in a part of the liver and are likely to grow or enlarge at site, and eventually they lead to damage to the liver and the whole body. This category comprises benign and malignant tumors, abscesses, cysts, and hydatidosis [53]. Complementary and alternative medicine is commonly used by hepatobiliary patients, more frequently by patients with unresectable malignant disease. The most common method is herbs or supplements, but prospective studies are needed to evaluate the outcomes [37]. 

## 3. Principles for Managing Liver Diseases

There are commonplace observations that we must make when any kinds of therapies are used to treat liver diseases [54]. The unique advantages of a particular therapy should also be noted [26]. Alternative and complementary medicine provides additional benefits to the patients with liver diseases who have been treated by conventional methods at the present time. Or it covers the shortages and compensates after the weaknesses of these methods. Choice of treatments must be evidence based and depends on the features of a certain category of liver diseases that the patient has [55]. 

Before treatment, evidence is to be collected to clarify that the diseased liver is altered in structure or function or both. Surgical and ablative therapies are indicated to liver diseases with structural abnormalities [56]. For all the patients with either structural or functional problems, medication is of paramount importance for a good outcome [57]. Special attention should be paid to nutritional and dietary therapies that are the constituents of complementary and alternative medicine. 

In selected conditions without contraindications, the first choice is invasive management for those suffering from tumors, cholelithiasis, congenital deformity of bile ducts, localized purulent infection, liver trauma, secondary stricture or obstruction of bile ducts, portal hypertension, and the Budd-Chiari syndrome. To reduce perioperative mortality and morbidity rate, minimally invasive operation and careful postoperative management are advocated [38]. Drainage and ablation can be used to treat cysts larger than 5 cm in diameter, but caution is advised in the puncture process as fluid escaping from hydatid cyst leads to fatal allergic shock of the patient [29]. 

When the interventional and medical therapies failed to cure the disease and the life expectancy of the patient is between 6 and 12 months, a liver transplant is possible. Recipients of liver transplantation include those suffering from congenital liver diseases, malignancy, end-stage cirrhosis, and acute or sub-acute liver failure [58]. Transcatheter arterial chemoembolization and radiofrequency ablation may be used as a bridge therapy for unresectable liver cancer to become resectable or transplantable [21]. Transjugular intrahepatic portosystemic stent shunting is used to treat portal hypertension in patients awaiting liver transplant [22].

Medication for the liver is used to provide specific treatment for the underlying liver disease, to supplement substances that are not absorbed well due to poor liver function, and to prevent complications or relieve symptoms [59]. Drugs are used to work against infectious organisms including viruses, bacteria, and parasites [60]. Others are used to decrease serum aminotransferase, relieve jaundice, reduce intoxication, or protect the hepatocyte plasma membrane [19]. 

Alternative and complementary medicine for liver diseases provides benefits by regulating immunity, controlling disease progression, improving quality of life, and prolonging survival. Some of the current management decisions are based on consensus and opinions, but evidence is needed [40]. 

## 4. Alternative and Complementary Medicine

There is a long history in the treatment of liver diseases of using traditional Chinese medicine which is considered a complementary or an alternative medical system in most Western countries [61]. Herbs as complementary and alternative medicine (CAM) are effective in the treatment of liver diseases, such as antagonizing fibrosis, steatosis, and hepatitis viruses, and in protecting the liver cell [62]. A recent investigation from Saudi Arabia assessed the prevalence of and attitudes towards the use of alternative medicine in liver disease patients. The results demonstrated that over half of the patients confessed the use of alternative and complementary medicine and over two-thirds of the patients thought that many health benefits exist in alternative and complementary treatments. Most of these studies demonstrated surprisingly common usage and a generally positive attitude toward the use [13]. However, apart from its beneficial effects, there are also undesirable and unfavorable that effects resulted from CAM. First of all, we should be aware of common adverse interactions between herbal/dietary substances and prescription medications. A prospective study from Korea investigated the incidence of herbal medicine-induced adverse effects on liver functions and concluded that herbs are rather safe when used alone, but the risk of adverse reactions may increase when herbs and conventional drugs are used together [63]. 

Alternative medicine means the use of CAM in place of conventional medicine. This approach avoids the risk of adverse effects when herbs and conventional drugs are taken concurrently [63]. Nonetheless, there has long been a hot debate about its entity. Some authors think that the term “alternative medicine” is a misnomer and cannot be clearly differentiated from “conventional medicine” [64]. Others argue that this kind of therapy is irrational, absurd, and should be rejected [65]. Complementary medicine refers to the use of CAM together with conventional medicine; thus, the risk of adverse reactions may increase as explained afore [63]. Integrated medicine is the combination of methods from conventional medicine and CAM, based on certain high-quality evidence of safety and effectiveness. The highlight of this approach is its concentration on evidence on essential factor for therapeutic decision-making. A recent study evaluated the safety and effectiveness of treating infantile cytomegalovirus hepatitis with integrated Chinese and Western medicine. The total effective rate was 95.0% in the group with integrated medicine, significantly higher than that (77.5%) in the group with conventional medicine alone [66]. Another study conducted interviews with ten leading experts in the field of CAM and integrative medicine in Western countries and concluded that integrated medicine can aid in removing barriers and opening up medical practice and research toward new visionary health care delivery and is concerned with changing conventional medical practice [67].

CAM does exist. The term “nonconventional medicine” in itself convinces us of the differences between CAM and conventional medicine. There is no reason to worry about the withering away of conventional medicine, but it is necessary to integrate it with other less expensive, and more holistic, more person-centered care [68]. Actually in 2007 alone, 83 million adults and 8.5 million children used CAM in the USA and paid about $34 billion in cash [69]. The widespread use of CAM in combination with Western drugs suggests that evidence-based research regarding the potential efficacy and safety of CAM methods is essential, especially when herbs are used in combination with Western drugs [70].

 Extracted active components of several herbs have been shown on the molecular basis for pharmaceutical effects against liver diseases, including curcumin, emodin, and quercetin [18, 20]. In addition, clinical trials revealed that these phytochemicals act as chemosensitizers through regulating the key players of the death receptor pathway and inhibiting DNA repair and apoptosis, showing anti-ancer effects [71]. 

One of the 27 institutes and centers that make up the National Institutes of Health (NIH) within the Department of Health and Human Services of the federal government of the United States, the National Center for Complementary and Alternative Medicine (NCCAM) conducted a review of alternative and complementary treatments in 2002 [23]. The most promising herbal treatment for hepatitis C was silymarin, an extract of milk thistle, a plant that has been used for centuries to treat liver disease and jaundice [31]. NCCAM also studied other alternative treatments of liver diseases. These include licorice root, ginseng, Schisandra, thymus extract, and colloidal silver. They concluded that the former three might possibly have positive effects on the liver, while the latter two were found to be ineffective in treating liver diseases [72, 73]. 

Use of CAM has been considerably increased in patients with liver disease. Herbs are the most common and widely used, treatment in traditional Chinese medicine. The application is diverse in clinical practice. For the treatment of liver diseases, there are single herb, herb pairs, groups of herbs, and fixed formulas [55, 74, 75]. Forms of herbal drug include ointment, pellet, ball, powder, and fluid decoction [76]. More standardized treatments are extracts from single herbs and formula injections, such as the Yinzhihuang injection, the Kuhuang injection, and the Tanreqing injection [77]. Take the Kuhuang injection as an example. This injection is derived from the famous formula Yinchenhaotang by Zhongjing Zhang (150 to 219 AD), a Han Dynasty physician and one of the most eminent Chinese physicians [24]. The main ingredients are capillary artemisia, Chinese rhubarb, *Sophora flavescens*, indigo woad leaf, and herba bupleurl. At present, both injection and concoction forms of this formula are prescribed for treatment of jaundice in thousands of hospitals in China and believed to be very effective in jaundice relief, decreasing aminotransferase, and hepatoprotection [27]. 

 However, both medical professionals and the patients should be more concerned about drug safety when herbal injection is used. Some patients who used herbal injection suffered adverse reactions, including death [77]. Here, things get very complicated. Quality of drugs and rational clinical administration are real problems at the present time. Falsified and substandard medicines are readily available in the private marketplace in low- and middle-income countries [78]. Insufficiency in detailed chemical analysis and research of related technologies on traditional Chinese medicine produces potential safety problems [77]. Even the standard and qualified herbal injections need pharmacovigilance and postmarketing safety monitoring. And there are problems that injections are misused and overused worldwide. Many injection treatments are unnecessary or could be given in an oral formulation. The behavior of patients and healthcare providers toward injection must be changed. Patients are inclined to injections because they think that they are stronger and faster cures. Doctors excessively prescribe injections because they believe that they please the patients well by so doing [79]. A special system should be set up to promote the rational use of herbal injections and supervise quality control of products and clinical application by acceptable worldwide guidelines [80, 81].  Table 1 demonstrates different treatments for various liver diseases provided by first-line clinical treatment in contrast to alternative treatment. 

## 5. Issues with Prescription for Liver Diseases

There are a variety of liver medicines. Unfortunately, few of them are actually used successfully in humans [82]. The liver has a burden of various tasks for the support of the whole body such as detoxification, lipid, protein, and carbohydrate metabolism, as well as bile and urea production, and it is the primary port of entry for ingested drugs. Drugs for liver diseases make this burden even heavier and may impose additional problems with the diseased liver which is the mainstay to metabolize the drugs taken in [9]. Attention must be paid to relieve its workload when prescription is given [83]. Drugs with documented adverse effects on liver functions and structures must be borne in mind and avoided. When the patient has comorbidities and additional drugs have to be used to treat ailments of other systems, caution is to be taken to prevent that; the use of those drugs may cause liver dysfunction or damage, especially drugs that are frequently encountered and may cause active chronic hepatitis, acute hepatic fatty infiltration, chronic cholestasis, cholestatic jaundice, dose-dependent or independent acute hepatitis, liver cirrhosis or fibrosis, liver granuloma, neoplasms, and vasculitis [12, 39, 43]. 

Regrettably, such agents are numerous and their adverse effects to the liver are often not recognized or neglected. For example, long use or large doses of acetaminophen, a common agent for fever and pain relief, cause active chronic hepatitis which can be antagonized by small thorowax herb or liquorice root [36, 50]. Coincidental use of agents containing salicylic acids exacerbates liver damage leading to lethal liver cell injury [84]. These agents include Chinese wingnut leaf in decoction for killing parasites, poplar for hepatitis and other digestive diseases, levigation of catkin (willow seeds) for jaundice, and decoction of holly for diarrhea [85, 86]. Even a number of fruits that are regarded as having very safe edibility may create liver problems when they are used excessively. Dry kiwi fruit is used for anorexia, dyspepsia, and hiccough [87], and tomato has been used to treat hepatitis [88]. Both of them are used as raw materials to extract industrial salicylic acids [89]. Chinese waxberry has been grown in China at for least 2000 years, and is used to treat digestive and inflammatory diseases. Recently, salicylic acids are identified and separated from this fruit, which accounts for the symptoms of nausea and vomiting resulted from excessive intake [90]. Another example is the damage to liver blood vessels caused by oral contraceptives [91], which can be made worse by coincidental intake of a large amount of vitamin A, contained in everyday consumption of animal viscera, eggs, milk products, and fruits [92]. 

Small amount or dosage is suggested for prescribing drugs for treatment of liver diseases. The essence of hepatoprotection is to reduce the liver's workload. Single agent, single remedy, or single drug prescriptions are encouraged [93]. When a compound recipe rather than a simple recipe must be used, the recommendation is the use of an empirical formula [94]. The simpler one is the better. Important origins of liver toxicity consist of adverse reaction to complementary medicines, over-the-counter medications, and prescription medications [12, 43, 95]. 

More words are provided here for reducing the liver's workload. Sometimes, we have to let the liver have more rest for reducing its workload and avoiding drug-drug reactions. No single potent drug is given to the patient but a prescription for easily digestible food. Glucuronolactone, vitamin C, and vitamin B complex can be used as supplements for the essential nutritional requirements for detoxifying functions of the liver [96, 97]. Adequate amounts of glucose and amino acids are needed for enriched nutrition for the liver. But too much glucose leads to diabetes, and to much fructose leads to fatty liver disease [5]. Inosine is necessary for cell repair and has been shown to have a therapeutic effect on leukocytopenia and thrombocytopenia [98]. Normal bowel movement is part of gastrointestinal well-being and beneficial for the gut microflora with the consequence of less absorption of toxins generated by intestinal bacteria [99]. 

In favor of the symbiotic relationship between the liver and intestinal tract, probiotic bacteria, for example, bifidobacteria, are better than antibiotic and prebiotic nutrition, say, nondigestible food ingredients [6]. As effects of drug-metabolizing enzyme systems on the pharmacological activity are more likely to occur in the intestine and liver, more attention should be paid to potential risk of nutrient-drug interactions to prevent undesired and harmful clinical consequences [100].

The liver depends for 80% of its blood supply on other organ systems [101]. So, bed rest is favorable for the nutrition and function of the liver by modifying its blood supply. The erect posture significantly decreases thee volume of blood flow to the liver up to 40% [102]. As the Chinese saying goes, “the liver stores blood.” This means on daytime more blood flows to the extremities, while at night it shifts to the liver. In the supine position, there is abundant blood in the liver. Excessive exercise is hazardous because the reduction of hepatic blood flow goes up to 80–85% when exercising, and chronic hepatitis leads to slow liver blood flow and increased viscosity [96]. Blood supply of the liver can be improved by proper use of red-rooted salvia and ligustici chuanxiong [103, 104]. 

Consumption of meat and fat intake is also linked to liver disease. Meat, especially red meat, is assumed to enhance the risk of cancer [105]. It is reported that red meat and saturated fat may be associated with increased risk of chronic liver diseases and liver cancer, whereas white meat may be inversely associated with that risk [106]. Nonetheless, meat consists of high concentrations and better bioavailability of folic acid, selenium, zinc, and other components that are asserted to be preventive candidates. Thus, a balanced diet is suggested that is rich in fruit and vegetables, containing meat and meat products in appropriate amount [105].

## 6. Recent Development

First do no harm. Beneficence, nonmaleficence, and self-government for the patient are substantial support for biomedical ethics that also adapt for the world of natural medicines including CAM for liver disease. Quality control of CAM methods and products is one of the greatest challenges that the professionals confront [62]. Hepatology has become an independent area with rapid expansion in some countries from a subspecialty of gastroenterology with doctors specializing as hepatologists [15]. A number of controlled prospective double-blind multicenter studies are ongoing with the newly discovered drugs with proven beneficial effects on animals [107, 108]. 

Structure and function of the liver are observed more precisely. The motion and deformation of the liver in real time within a region of interest can be measured by a real-time 3D image guidance system with a seeker needle including embedded electromagnetic tracking sensors [10]. This facility is useful in radiofrequency ablation and other percutaneous ablative procedures for treatment of space-occupying lesions. Tests are developed of various antibodies for autoimmune liver diseases that have overlapping features in clinical, histological, radiological, and serological manifestations, and currently randomized and controlled outcome data are insufficient to develop standardized diagnostic criteria [40]. 

Acceptable effects have been obtained on researches in treatment with alternative and complementary medicine of chronic liver diseases in the aspects of idiopathic hepatitis, nonalcoholic steatohepatitis, liver fibrosis, oxidative stress, and immune regulation. Adiponectin is shown to play a role in the suppression of the metabolic derangements that may result in nonalcoholic fatty liver disease. Leptin is revealed to play a key role in regulating energy intake and energy expenditure, including appetite and metabolism [5]. The combination use of adiponectin and leptin has been shown to completely reverse insulin resistance associated with obesity [109]. Herbs that increase adiponectin and/or leptin include Chinese dong quai root, goldenseal root, bacopa, green tea, and milk-vetch [104, 110, 111]. 

Trace elements serve critical roles in normal liver function and liver disease. However, excessive trace elements are commonly toxic to the liver. Selenium deficiency in hepatocytes leads to cell death by oxidative stress. Foods rich in selenium comprise nuts, sunflower seeds, shellfish, and liver [105]. A number of herbs are also high in selenium, for example, garlic, ginger, mustard seeds, fenugreek, and chervil [23, 112]. Other important trace elements with respect to liver diseases include iron, copper, zinc, calcium, magnesium, and manganese [48, 105]. 

Extensive and in-depth researches of CAM are needed to accumulate evidence of defined therapeutic effects. Although licorice root and ginseng have independent effects on liver cancer, the combinations of both extracts were found to have an antagonistic effect on cell viability and increased cultured Hep-G2 survival [113]. 

Systematic analyses are conducted of the therapeutic effects of the working mechanisms of some formulas using immunohistochemistry, biochemistry, metabolomics, and proteomics [114]. Chronobiology emerges with the current knowledge of circadian rhythms in Hepatology. A certain drug can be therapeutic and safe when taken at one time, but insufficient or poorly tolerated at another. Circadian rhythm abnormalities are associated with a variety of types of chronic liver diseases including hepatitis C, primary biliary cirrhosis, Wilson disease, and fibrosis and hepatic encephalopathy [115].

## 7. Conclusions

There is a vast body of knowledge ever increasing about the treatment of liver disease with alternative and complementary medicine for which hundreds of thousands of literatures have been documented. Liver disease has a variety of presentations and causes a great worldwide public health problem which threatens the wellness of billions of people. Incidences of many types of liver disease are currently rising. Although a debate exists about its entity, alternative and complementary medicine is widely used and it is improving, and it covers the shortages and compensates for the weaknesses of conventional methods in the treatment of liver disease. Alternative and complementary medicine for liver diseases provides benefits by regulating immunity, controlling disease progression, improving quality of life, and prolonging survival. Some of the current management decisions are based on consensus and opinions, but evidence is needed to confirm the effects of single herb, herb pairs, groups of herbs, and fixed formulas. 

Drug safety is concerned especially when herbal injection is used. Even the standard and qualified herbal injections need pharmacovigilance and postmarketing safety monitoring. A special system should be set up to promote the rational use of herbal injections and supervise quality control by acceptable worldwide guidelines. When CAM is used to treat liver disease, a manner of single agent, simple method, and small amount is suggested to reduce the liver's workload. Attention should also be paid to the nutrition and blood supply of the liver, as well as drug-food interactions. 

Recent development includes researches in quality control of CAM methods and products, emerging of specialists such as hepatologists, more precise observation of the structure and function of the liver, acceptable effects on several liver diseases, importance of trace elements, and hepatic chronobiology. Studies are encouraged such as controlled prospective double-blind multicenter studies with the newly discovered drugs with proven beneficial effects on animals.

New aspects that CAM can provide include meditation, support groups, and integrative medicine. More and more individuals are using CAM for treatment of liver diseases, and most of them believe that CAM has numerous health benefits. The growing public interest in CAM reflects the fact that CAM is now widely accepted as an important ally in our fight against liver diseases. However, many widely used CAM treatments remain untested. For example, St. John's wort which has been used as a treatment of depression from liver cancer is now the number-one-selling nutritional supplement in the USA. But it has not been rigorously evaluated and found with untoward effects on HIV drugs, transplanted organs, and sunshine in recent studies. So it is urgent to collect compelling and rigorous data of CAM treatments. The public needs to be kept well informed to reject readily modalities that are found unsafe or ineffective. 

## Figures and Tables

**Table 1:** Treatments of various liver diseases provided by first line clinical treatment in contrast to alternative treatment.

Diseases/stages/conditions	First line treatment/conventional treatment	Complementary and alternative medicine	Mechanism
Alcoholic liver diseases	Nutritional therapy, maybe parenteral [14, 15]	Enteral nutritional supplementation [14, 15]	Combating malnutrition
Autoimmune liver diseases	Prednisone alone or with azathioprine [16, 17]	Curcumin [18–20]	Modulating biological activity of signaling molecules
Cirrhosis	Surgery/transjugular intrahepatic portosystemic shunts [21, 22]	Silymarin [23], Yinchenhao Tang, and Xaiocaihu Tang [24]	Hepatoprotective, directly affecting hepatitis virus
Congenital liver diseases	Combined surgery or hybrid operation [25], gene therapy [26]	Huangqi tang [24], the Kuhuang injection [27]	Hepatoprotective, relieving jaundice
Infectious liver diseases	Antimicrobial agents, invasive [7, 28, 29]	Probiotics, silymarin [30, 31]	Anti-inflammatory and selective antimicrobial effects
Intoxicated liver diseases	Chelation therapy, supportive care [32, 33]	Essential phospholipids from soybean [34]	Detoxification
Metabolic liver diseases	Diet, nutritional supplementation [35]	Glycyrrhizin and matrine [36]	Hepatoprotective and nonspecific anti-inflammatory effect
Space-occupying liver diseases (tumors)	Invasive (surgery, ablation, transplantation) [10, 37, 38]	Phytochemicals, curcumin, emodin, and quercetin [18, 20]	Death receptor pathway, inhibiting DNA repair and apoptosis
